# P-2138. The Significance of Low Titer Serum Cryptococcal Antigen Testing from 2017 to 2023

**DOI:** 10.1093/ofid/ofae631.2293

**Published:** 2025-01-29

**Authors:** Jenna Wilson, Julie A Ribes, Mark Irwin, Thein Myint

**Affiliations:** University of Kentucky College of Medicine, Lexington, Kentucky; University of Kentucky, Lexington, KY; University of Kentucky, Lexington, KY; University of Kentucky, Lexington, KY

## Abstract

**Background:**

In the fall of 2016, a cluster of low positive serum cryptococcal antigen titers (SCrAg) ≤ 1:20 was traced back to a manufacturing defect. Subsequently, SCrAg ≤ 1:10 were reported with a disclaimer to correlate clinically at our institution. This study aimed to determine the performance of the assay since 2017, after the test improvement.

**Figure:**
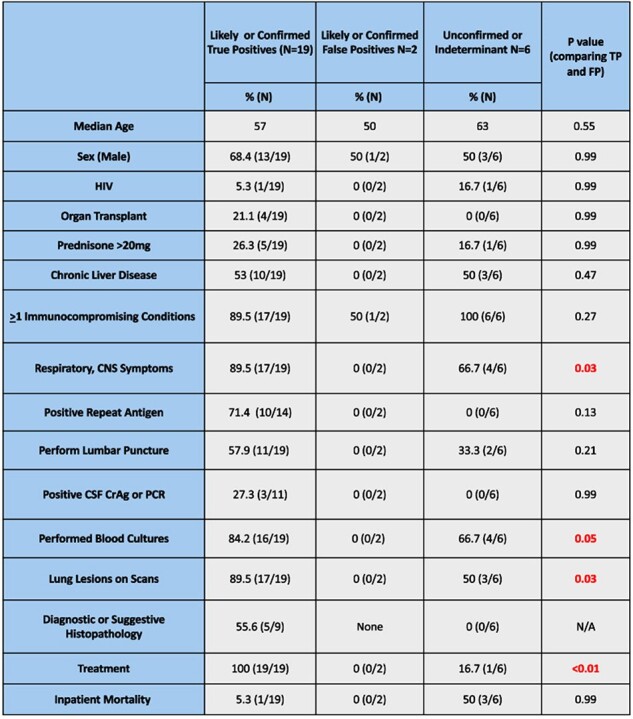

Comparison of Baseline Characteristics, Laboratory Findings and Outcome Among Three Groups

**Methods:**

This was a retrospective study of patients with first time low titer SCrAg (≤1:20) tested at UK HealthCare from Jan 2017-Oct 2023. Patients were classified as true positives (TP), likely false positive (FP) and indeterminate based on repeat SCrAg, PCR or culture (CSF, Blood, respiratory, other), histology, correlating symptoms and/or detection of nodular infiltrates on CT, all extracted via chart review from the electronic medical record.

**Figure:**
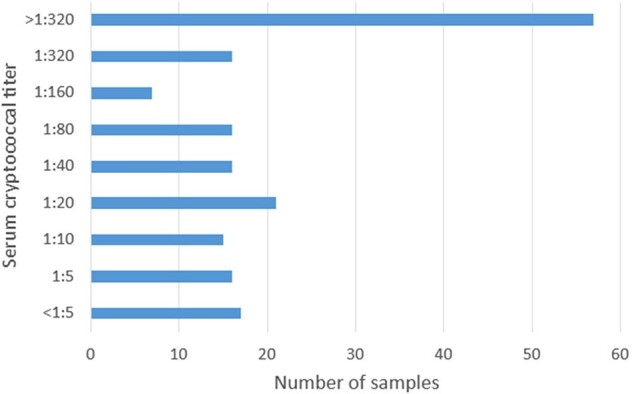

Number of Positive Serum Cryptococcal Antigen Samples Based on Titers​

**Results:**

Of 3716 SCrAg performed during the study, 181 (4.9%) were positive. 69/180 (38%) positive samples were SCrAg ≤1:20. We excluded 32 TP tests due to prior higher SCrAg or prior history of cryptococcal meningitis and focused on the significance of newly identified low titer patients. 37 (0.9%) low titer SCrAg samples were seen in 27 patients. 19/27 patients (70.4%) were classified as TP, 2 (7%) FP, and 6 (22.2%) were classified as indeterminate (Table 1). Lumbar puncture (LP) was performed in 57.9% (11/19) of those classified as TP, and of those, 27.3% (3/11) had confirmed cryptococcal meningitis. Five of the nine patients that had histologic examination demonstrated organisms consistent with cryptococcosis. Antifungal therapy was administered in 100% (19/19) TP compared to 0% (0/2) FP (p < 0.01).

**Conclusion:**

False positive low titer SCrAg are not frequent. When newly detected, these low titer positives are associated with additional corroborating evidence suggesting cryptococcosis. Patients with likely true positive SCrAg had respiratory and CNS symptoms and abnormal imaging more often than those with FP SCrAg. Low positive SCrAg should be extensively investigated for evidence of cryptococcal infection.

**Disclosures:**

All Authors: No reported disclosures

